# Risk Factors for Respiratory Depression Associated with Tramadol Based on the Global Pharmacovigilance Database (VigiBase)

**DOI:** 10.3390/ph17020205

**Published:** 2024-02-05

**Authors:** Sunny Park, Geon-Ho Lee, Soyun Kim, Solee Kim, Yeju Kim, Soo-An Choi

**Affiliations:** 1 College of Pharmacy and Research Institute of Pharmaceutical Sciences, Korea University, Sejong 30019, Republic of Korea; psunny0708@korea.ac.kr; 2 College of Pharmacy, Korea University, Sejong 30019, Republic of Korea

**Keywords:** analgesics, hypoventilation, tramadol, pharmacovigilance, World Health Organization, VigiBase

## Abstract

Tramadol, a weak μ-opioid receptor agonist, has been used worldwide for pain management. It is considered to have a favorable safety profile without serious adverse events; however, safety issues of respiratory depression were proposed by regulatory governments. We aimed to examine the risk and contributing factors associated with tramadol-related respiratory depression using a real-world database, VigiBase. Disproportionality analysis of tramadol and tramadol/paracetamol was performed using proportional reporting ratios, reporting odds ratios, and information components for all drugs and opioids. Factors related to respiratory depression, including sex, age, presence of abuse, death, and various concomitant medications, were evaluated. Among 140,721 tramadol reports, respiratory depression was reported in 1126 cases, 81.3% of which were deemed serious. Five adverse events were detected as signals of tramadol-related acute central respiratory depression (ACRD) in 882 reports. A higher proportion of ACRD cases in children and adolescents was observed than all adverse events cases of tramadol. Concomitant users of CYP2D6 inhibitors, opioids, benzodiazepines, and anti-depressant drugs showed a higher proportion in ACRD cases than non-ACRD cases. ACRD was related to drug abuse and death. This pharmacovigilance study, using VigiBase, confirmed a high risk of respiratory depression (a serious, potentially fatal adverse event) secondary to the use of tramadol, especially in pediatric patients, drug abusers, or during concomitant use of opioids, benzodiazepines, or antidepressants.

## 1. Introduction

Tramadol is a widely used analgesic with a dual mechanism of action: a weak agonist at the μ-opioid receptor, and an inhibitor of serotonin (5-HT) and norepinephrine reuptake [1,2]. The predominant tramadol-induced adverse reactions include confusion, sleepiness, nausea, vomiting, seizures, dizziness, dry mouth, sedation, and headaches [3]. At therapeutic doses, tramadol reportedly exhibits a favorable safety profile without serious adverse events (AEs) as seen in pure opioids, such as morphine [1]. Moreover, tramadol has been associated with certain advantages, including easy and wide availability and low abuse liability [4].

Globally, the use of tramadol has increased rapidly over the past few decades, resulting in a 22.8% growth between 2012 and 2015 in the United States, making tramadol one of the most prescribed opioids with excessive expectations of safety and low abuse liability [5,6]. However, concomitant use of medications that increase serotonin level, such as fluoxetine, venlafaxine, or overuse of tramadol, has been found to be associated with serious AEs, such as serotonin syndrome or seizures [7,8,9,10]. Respiratory depression, which is potentially life-threatening and causes substantial morbidity and mortality [11], has been proposed in specific patient populations, such as morbidly obese patients with normal renal function [12] and those with renal impairment and cytochrome P450 family 2 subfamily D member 6 (CYP2D6) gene duplication [13]. 

In children, tramadol’s labeled indication varies from country to country. In Europe, tramadol is approved for use in children over 1–3 years of age, depending on the country, for management of moderate to severe nociceptive pain. In the United States, tramadol is approved only for children over 17 years of age, but appears to be used regularly nonetheless [14,15]. In 2017, the US Food and Drug Administration (FDA) strengthened drug labels for tramadol to highlight the possibility of breathing problems in children [16]. Health Canada also announced the potential of respiratory depression in children and adolescents, and recommended avoiding the use of tramadol in patients less than 18 years of age [17]. The European Medicines Agency (EMA) restricts the use of tramadol for postoperative pain management in children with certain conditions, such as obstructive sleep apnea (OSA) and compromised respiratory function [18,19].

Respiratory depression is a well-known adverse effect associated with opioid administration, mediated via μ-opioid receptors [20], especially in cases of abuse or overdose and when combined with sedatives or illicit substances [21]. Respiratory depression, also known as hypoventilation, is the primary cause of opioid-induced death [22]. Additionally, a study reported apnea in 3.6% of patients with tramadol abuse or self-addiction, regardless of risk factors [23]. However, most previous studies of tramadol-related respiratory depression were based on case reports or series with a limited number of patients. 

Real-world databases have accumulated post-marketing safety data related to tramadol [24] since it was first marketed in Germany in 1977. Studies using real-world databases become important in providing evidence of effectiveness and safety in clinical practice [25]. Analysis of spontaneous reports of suspected adverse drug reactions (ADRs) is a valuable tool in the detection of previously unknown drug adverse reactions. According to the FDA, their new warning in 2017 was based on nine cases of respiratory depression, including three deaths reported in children under 18 years of age between 1969 and 2016 to the FDA Adverse Event Reporting system [16]. Several pharmacovigilance studies assessing tramadol have been reported [5,26]; however, no previous study has investigated tramadol-related respiratory depression using a VigiBase. Therefore, it is important to evaluate the risk and factors for respiratory depression of tramadol based on a large, real-world database. Herein, we aimed to identify tramadol-related respiratory depression using a real-world global database, and to determine factors for risk of respiratory depression of tramadol.

## 2. Results

### 2.1. Demographic Characteristics of Safety Reports

Of the 23,811,236 cases reported to VigiBase through January 3, 2021, tramadol and tramadol/paracetamol were identified in 140,721 (0.59%) and 51,401 (0.22%) reports, respectively. The acute central respiratory depression (ACRD) cases accounted for 1126 (0.8%) of the 140,721 reports for tramadol. Tramadol/paracetamol showed ACRD in 108 cases (0.2%) of the 51,401 reports. Overall, AEs and ACRD reports were predominant among women. Although the majority of all AEs were reported by other healthcare professionals, physicians were the most frequent reporters of ACRD. The majority of the cases were reported from Asia, while ACRD reports were from the Americas and Europe (Table 1). No significant quantitative changes have been identified in safety reporting trends since 2017, when the tramadol warning was issued. Supplementary Table S1 presents 938 and 94 serious cases related to tramadol and tramadol/paracetamol, respectively. 

### 2.2. Disproportionality Analysis 

Tramadol demonstrated five positive signals for ACRD over all of the other reported drugs, with “Respiratory arrest”, “Respiratory depression”, “Bradypnoea”, “Hypoventilation”, and “Respiratory rate decreased in 882 reports.” On the other hand, tramadol/paracetamol only showed “Bradypnoea” as a signal. For opioids, no signal was detected for tramadol or tramadol/paracetamol. Table 2 presents the disproportionality analysis results over all drugs and opioids. In particular, when an additional stratification analysis was performed, the same signals were detected in children under 18 years of age, except for “reduced respiratory rate” (Supplementary Table S2).

### 2.3. Factors Related to ACRD of Tramadol 

Of 140,721 tramadol reports, there were 1126 (0.8%) ACRD cases. Compared to non-ACRD cases, males and patients under 17 years were predominant among ACRD cases. Most ACRD cases were reported from America by physicians, while non-ACRD cases were from Asia and reported by consumer or non-healthcare professionals. Concomitant users of CYP2D6 inhibitors, opioids, benzodiazepines, and anti-depressant drugs showed a higher proportion in ACRD cases than non-ACRD cases. ACRD cases were related to drug abuse and death. (Table 3). It was confirmed that 19.8 to 31.1% of ACRD patients were taking drugs that increased the risk of respiratory depression, which was 4.4 to 7 times higher than the non-ACRD group. 

## 3. Discussion

Herein, disproportionality analysis identified tramadol-related respiratory depression with several signals over all drugs, suggesting that risk of respiratory depression of tramadol cannot be overlooked. On the other hand, there was no signal over opioids, suggesting that tramadol seems to have a lower risk of ACRD than opioids. These findings can be attributed to the induction of respiratory depression via μ-opioid receptors [27,28], and the weak opioid agonistic activity of tramadol. Older studies demonstrated the lack of correlation between tramadol and respiratory depression [29,30]. However, tramadol has been used concomitantly with various drugs, as shown in this study, because tramadol is considered safe. Concomitant use of tramadol with other drugs, including selective serotonin reuptake inhibitors, benzodiazepines, or first-generation antihistamines, was reported to increase serotonin syndrome risk [31]. Using real-world data, our study confirmed that respiratory depression after tramadol use is consistent with the findings of certain previous reports [12,23]. Thus, the risk of tramadol-related respiratory depression should be carefully monitored despite there being a lower risk than opioids.

All AEs including non-ACRD were more frequently reported in Asia, whereas AEs related to ACRD were more frequently reported in the Americas and Europe. This could be due to genetic differences, including CYP2D6 polymorphiams [18]. A higher proportion of ACRD cases (9.9%) in children and adolescents was observed than all AEs cases (2.7%) of tramadol. In addition, patients under 17 years old showed a higher proportion of ACRD cases than non-ACRD cases related to tramadol, suggesting a high risk of respiratory depression in children and adolescents, as reported by the FDA and Health Canada [16,17]. 

An overdose of tramadol may relate to respiratory depression, as reported previously [8,9,32]. Herein, tramadol/paracetamol-related respiratory depression was detected as a signal only in bradypnoea, contrary to tramadol. This finding suggests that combined tramadol/paracetamol formulations comprise a lower amount of tramadol than tramadol alone, and respiratory depression is a dose-dependent AE, as reported in previous studies [8,28]. Similar to these results, risk of respiratory depression, the major cause of death in opioid addicts [33], was higher in drug abusers at high risk of overdose.

Our results suggest that concomitant use of opioids, benzodiazepines, or antidepressant drugs may increase the risk of respiratory depression. Opioids and benzodiazepines are well-known to cause respiratory depression [20,34]. Antidepressant drugs that increase serotonin levels [2,35] can lead to serotonin syndrome when administered concomitantly with tramadol. Serotonin syndrome includes seizures, rhabdomyolysis, metabolic acidosis, acute respiratory distress syndrome, and respiratory failure [36]. In our study, reports of ACRD were very few, accounting for only 0.8% of the total AEs reported with tramadol. However, it was confirmed that 19.8 to 31.1% of ACRD patients were taking drugs that increased the risk of respiratory depression, which was 4.4 to 7 times higher than the non-ACRD group. This ultimately appears to have contributed to the higher mortality rate (12.6%, 8.6 times) in the ACRD group compared to the non-ACRD group. Although it is clear that ACRD is a fatal adverse reaction, this study shows that already known risk factors have not yet been considered in real-world settings, so we believe that more vigilance is needed.

Tramadol is primarily metabolized to O-desmethyltramadol (M1) by CYP2D6. The M1 metabolite has a markedly higher affinity for the µ-opioid receptor than tramadol. Thus, in individuals with increased CYP2D6 activity, standard tramadol doses may increase the risk of AEs, owing to enhanced exposure to M1 [18]. The highly polymorphic CYP2D6 gene is one of the most investigated CYPs with regard to genetic polymorphisms [37]. Genetic differences in metabolic enzymes could underlie the risk of respiratory depression [13]. There is a large interindividual variability in the enzyme activity of CYP2D6 within a population and between ethnic groups. The prevalence of the CYP2D6 ultrametabolizer varies, but is thought to occur in approximately 1–10% of Caucasians (European, North American), 3–4% of African Americans, and 1–2% of East Asians (Chinese, Japanese, and Korean) [38]. Specifically, approximately 40% of the United States population is expected to carry one of the “extreme phenotype”, i.e., to be a poor- or ultra-metabolizer [39]. Therefore, the FDA-approved drug label warns that ultra-rapid metabolizers should avoid tramadol, owing to the risk of life-threatening respiratory depression and signs of opioid overdose (e.g., extreme sleepiness, confusion, or shallow breathing), unlike the EMA [40]. Although our study could not determine genetic polymorphisms, owing to the absence of genetic data, the relatively frequent reporting from America and Europe cannot rule out these effects. 

Contrary to this result, a higher proportion of respiratory depression was observed in patients administered concomitantly with CYP2D6 inhibitors. CYP2D6 inhibition has been shown to result in clinically significant failure of tramadol bioactivation with a significant reduction of analgesic opioid efficacy, such as hydrocodone [41,42]. However, in the case of tramadol, the results are more complicated due to its dual mechanism of action. CYP2D6 inhibition not only reduces the formation of M1, it also increases tramadol parent drug plasma concentrations, which may be associated with an increased risk of the potentially life-threatening, dose-dependent serotonin syndrome [43]. Therefore, there is the possibility of respiratory depression caused by serotonin syndrome, but confounders contributing to respiratory depression, which cannot be considered in this study, may exist in CYP2D6 inhibitor concomitant users. Therefore, further studies should be conducted.

This study has several limitations owing to spontenous reporting data. Tramadol is currently available in various dosages and forms, such as tablets, oral drops, solutions for injection, and suppositories [44], exhibiting different pharmacokinetic characteristics; however, we did not analyze the dosage and formulation of tramadol due to limited information. Genetic differences between the patients could not be determined, which could have impacted the pharmacokinetics of tramadol. Additionally, VigiBase provides little information to identify temporal relationships with events unrelated to drug use (e.g., operation) or clinical laboratory data. Nevertheless, we confirmed serious or life-threatening tramadol-related respiratory depression with various exposures in the real-world, despite its well-known safety and regulatory recommendation for careful use of tramadol due to high risk of respiratory depression; hence, this study is valuable. In addition, this is the first study to evaluate factors of tramadol-related respiratory depression based on a real-world global database.

## 4. Materials and Methods

### 4.1. Data Source and Ethical Statement

The present study was performed using individual case safety reports (ICSRs) for tramadol and tramadol/paracetamol from the World Health Organization Uppsala Monitoring Center (WHO-UMC) VigiBase. The data included age group, sex, reporter, date, the continent of the primary source, name of the drug used, AEs, and seriousness reported by members participating in the WHO International Drug Monitoring Program from 1968 to January 3, 2021. Serious AEs are defined by the US FDA as those that result in death, life-threatening conditions, hospitalization (initial or prolonged), disability or permanent damage, or a congenital anomaly or birth defect requiring intervention to prevent permanent impairment or damage [22]. The ICSRs were reported by local physicians, pharmacists, other healthcare providers, and the public. We analyzed the ICSRs of all reports and respiratory depression reports associated with tramadol and tramadol/paracetamol. This study was approved by the Institutional Review Board of Korea University, which waived the requirement for informed consent owing to the use of secondary data (IRB No. 2022–0097). Data might be used after approval is obtained from the UMC at https://who-umc.org/ (accessed on 1 March 2023) (request numbers ER007-2021 and ER-026-2021). The datasets analyzed are not publicly available because of the ongoing collection of AE reports. However, they are available from UMC upon reasonable request and order for academic purposes via the previously mentioned website. Therefore, the request number means that our order and approval have been completed and the available dataset has been provided. All procedures were performed in accordance with the relevant guidelines and regulations. The database does not contain sensitive information that could identify an individual.

### 4.2. Disproportionality Analysis and Signal Detection Criteria

Reports of AEs associated with a drug are not necessarily true ADRs, that is, they may be temporally associated with a drug but not caused by the drug. Hypothesis generation of new possible side effects from such data is referred to as signal detection, a signal being defined by the WHO as: “Reported information on a possible causal relationship between an AE and a drug, the relationship being unknown or incompletely documented previously.” [45]. A two-by-two table was generated to evaluate disproportionality, a method utilized as a basic approach for detecting signals in large databases (Table 4). The most frequently used disproportionality indices, the proportional reporting ratio (PRR), reporting odds ratio (ROR), and information component (IC) [46,47,48,49] were calculated based on cases reported as suspicious or interacting. The IC was developed and validated by the UMC; it relies on a Bayesian confidence propagation neural network and the formula is as follows:

$$IC=log2\frac{N_{observed}+0.5}{N_{expected}+0.5}$$

in which $N_{expected}$ is estimated by $N_{expected=\frac{N_{drug}\times N_{effect}}{N_{total}}}$, $N_{drug}$ is the total number of reports involving the drug studied, and $N_{effect}$ is the total number of reports for the adverse events, regardless of drug.

Due to well-known, opioid-induced respiratory depression, disproportionality analysis was performed over all drugs as well as opioids to examine the risk of respiratory depression of tramadol. Opioids were defined as drugs with the Anatomical Therapeutic Chemical codes N02A and N01AH. Considering events reported at least three times, PRR and ROR ≥ 2 and a lower limit of the 95% confidence interval of IC ≥ 0 indicated positive signals, as shown in Table 5. To identify the risk of tramadol-related respiratory depression in pediatric patients, we performed additional disproportionality analysis using stratification by age.

### 4.3. Standardized Medical Dictionary for Regulatory Activities Query and the Definition of Respiratory Depression

The Medical Dictionary for Regulatory Activities (MedDRA) terminology, the global standard for recording AEs and medical histories [25], was used in the present study. The Standardized Medical Dictionary for Regulatory Activities Query (SMQ), a validated and predetermined set of MedDRA terms [26], was employed to group terms related to respiratory depression. Respiratory depression was defined as “acute central respiratory depression” (ACRD) of the SMQ in a narrow scope, including the following factors: “acute respiratory distress syndrome”, “acute respiratory failure”, “apnoea”, “apneic attack”, “apparent life threatening event”, “bradypnoea”, “breath holding”, “breath sounds abnormal”, “hypopnea”, “hypoventilation”, “infantile apnoea”, “neonatal respiratory arrest”, “neonatal respiratory depression”, “respiratory arrest”, “respiratory depression”, “respiratory depth decreased”, “respiratory failure”, “respiratory paralysis”, and “respiratory rate decreased.”

### 4.4. Factors Related to Respiratory Depression of Tramadol

The factors related to respiratory depression of tramadol, including sex, age, presence of abuse, death, and various concomitant medications with tramadol, were evaluated. CYP2D6 inhibitors were selected using the FDA’s examples of drugs that interact with CYP enzymes and transporter systems [27]. Concomitant medications of opioids, benzodiazepines, and anti-depressant drugs included medications concomitantly reported in more than 20 cases with tramadol. Drug abuse was defined by cases reported with AEs included in “Drug abuse and dependence” of SMQ. A chi-square test was performed to determine the association between ACRD and factors, using SAS version 9.4. For variables with more than three categories, the next step was to perform a post hoc test to find out which categories in the contingency table differ from the expected values.

## 5. Conclusions

This pharmacovigilance study using real-world data from the Uppsala Monitoring Center confirmed a high risk of respiratory depression (a serious, potentially fatal adverse event) secondary to the use of tramadol, especially in pediatric patients, drug abusers, or during concomitant use of opioids, benzodiazepines, or antidepressants.

## Figures and Tables

**Table 1:** Demographics of the total and respiratory depression related reports of tramadol and tramadol/paracetamol on VigiBase.

	All Cases of Tramadol (n = 140,721)	ACRD Cases of Tramadol (n = 1126)	All Cases of Tramadol /Paracetamol (n = 51,401)	ACRD Cases of Tramadol/Paracetamol (n = 108)
Sex (N, %)				
Male	50,077 (35.6%)	438 (38.7%)	15,571 (30.3%)	44 (40.7%)
Female	85,677 (60.9%)	589 (52.6%)	34,781 (67.7%)	60 (55.6%)
Not known	4967 (3.5%)	99 (8.7%)	1049 (2.0%)	4 (3.7%)
Age				
≤11 years	1172 (0.8%)	67 (6.0%)	142 (0.3%)	0 (0.0%)
12–17 years	2650 (1.9%)	44 (3.9%)	464 (0.9%)	5 (4.6%)
18–44 years	38,203 (27.2%)	350 (31.1%)	8734 (17.0%)	27 (25.0%)
45–64 years	45,481 (32.3%)	274 (24.3%)	18,596 (36.2%)	22 (20.4%)
65–74 years	19,300 (13.7%)	96 (8.5%)	10,071 (19.6%)	18 (16.7%)
≥75 years	16,896 (12.0%)	117 (10.4%)	9069 (17.6%)	20 (18.5%)
Unknown	17,019 (12.1%)	178 (15.8%)	4325 (8.4%)	16 (14.8%)
Reporter				
Consumer/Non-healthcare professional	17,818 (19.8%)	113 (10.0%)	11,483 (22.3%)	9 (8.3%)
Physician	37,733 (26.8%)	498 (44.2%)	10,458 (20.4%)	64 (59.3%)
Other healthcare professional	44,831 (31.9%)	232 (20.6%)	16,375 (31.9%)	15 (13.9%)
Pharmacist	15,237 (10.8%)	160 (14.2%)	11,794 (23.0%)	16 (14.8%)
Lawyer	721 (0.5%)	13 (1.2%)	15 (0.03%)	0 (0.0%)
Unknown	14,381 (10.2%)	110 (9.8%)	1276 (2.5%)	4 (3.7%)
Serious cases	25,562 (65.8%)	938 (83.3%)	5047 (9.8%)	94 (87.0%)
Region				
Americas	25,061 (17.8%)	567 (50.4%)	1319 (2.6%)	34 (31.5%)
Europe	25,994 (18.5%)	437 (38.8%)	7018 (13.7%)	42 (38.9%)
Asia	86,202 (61.3%)	86 (7.6%)	42,846 (83.4%)	31 (28.7%)
Oceania	2363 (1.7%)	31 (2.8%)	6 (0.01%)	0 (0.0%)
Africa	1101 (0.8%)	5 (0.4%)	212 (0.4%)	1 (0.9%)
Year				
≤2013	35,328 (25.1%)	502 (44.6%)	7515 (14.6%)	36 (33.3%)
2014	12,671 (9.0%)	127 (11.3%)	4640 (9.0%)	22 (20.4%)
2015	13,902 (9.9%)	79 (7.0%)	5437 (10.6%)	7 (6.5%)
2016	11,554 (8.2%)	70 (6.2%)	3517 (6.8%)	14 (13.0%)
2017	15,894 (11.3%)	56 (5.0%)	7925 (15.4%)	9 (8.3%)
2018	15,590 (11.1%)	105 (9.3%)	7310 (14.2%)	8 (7.4%0
2019	15,615 (11.1%)	89 (7.9%)	6594 (12.8%)	8 (7.4%)
≥2020	20,167 (14.3%)	98 (8.7%)	8463 (16.5%)	4 (3.7%)

The cases included suspect and interacting reports.

**Table 2:** Disproportionality analysis outcomes associated with acute central respiratory depression.

Adverse Events	Tramadol (n = 140,721)							Tramadol/Paracetamol (n = 51,401)						
	No. of Reports *	Above Full Database			Above Opioids			No. of Reports	Above Full Database			Above Opioids		
		PRR (CI)	ROR (CI)	IC_025_	PRR	ROR	IC_025_		PRR (CI)	ROR (CI)	IC_025_	PRR (CI)	ROR (CI)	IC_025_
Respiratory arrest	358	2.91 (2.62–3.23)	2.92 (2.63–3.24)	1.37	0.27 (0.25–0.30)	0.27 (0.24–0.30)	−1.83	13	0.29 (0.17–0.49)	0.29 (0.17–0.49)	−2.66	0.03 (0.02–0.05)	0.03 (0.02–0.05)	−5.85
Respiratory depression	357	5.52 (4.97–6.13)	5.53 (4.98–6.15)	2.26	0.36 (0.32–0.40)	0.36 (0.32–0.40)	−1.46	35	1.44 (1.04–2.01)	1.45 (1.04–2.01)	0.003	0.10 (0.07–0.14)	0.10 (0.07–0.14)	−3.07
Respiratory failure	129	0.56 (0.47–0.67)	0.56 (0.47–0.67)	−1.08	0.40 (0.33–0.48)	0.40 (0.33–0.48)	−1.42	20	0.24 (0.15–0.37)	0.24 (0.15–0.37)	−2.73	0.18 (0.12–0.28)	0.18 (0.12–0.28)	−3.07
Bradypnoea	73	8.93 (7.06–11.3)	8.93 (7.06–11.3)	2.67	0.44 (0.35–0.56)	0.44 (0.35–0.56)	−1.38	10	3.21 (1.73–5.99)	3.21 (1.73–5.99)	0.50	0.17 (0.09–0.32)	0.17 (0.09–0.32)	−3.42
Apnoea	69	0.62 (0.49–0.78)	0.62 (0.49–0.78)	−1.05	0.18 (0.14–0.23)	0.18 (0.14–0.23)	−2.60	6	0.15 (0.04–0.33)	0.15 (0.04–0.33)	−4.04	0.05 (0.02–0.11)	0.05 (0.02–0.10)	−5.58
Hypoventilation	60	2.19 (1.70–2.83)	2.19 (1.70–2.83)	0.72	0.26 (0.20–0.33)	0.26 (0.20–0.33)	−2.14	4	0.40 (0.15–1.06)	0.40 (0.15–1.06	−2.97	0.05 (0.02–0.14)	0.05 (0.02–0.14)	−5.79
Respiratory rate decreased	34	2.22 (1.58–3.11)	2.22 (1.58–3.11)	0.59	0.18 (0.13–0.25)	0.18 (0.13–0.25)	−2.76	7	1.24 (0.59–2.61)	1.24 (0.59–2.61)	−0.97	0.11 (0.05–0.24)	0.11 (0.05–0.24)	−4.25
Acute respiratory failure	33	0.68 (0.48–0.96)	0.68 (0.48–0.96)	−1.08	0.56 (0.39–0.80)	0.56 (0.39–0.80)	−1.26	7	0.39 (0.19–0.83)	0.39 (0.19–0.83)	−2.54	0.33 (0.16–0.71)	0.33 (0.16–0.71)	−2.71
Hypopnoea	29	1.91 (1.32–2.75)	1.91 (1.32–2.75)	0.33	0.51 (0.35–0.74)	0.51 (0.35–0.74)	−1.42	4	0.72 (0.27–1.91)	0.72 (0.27–1.91)	−2.17	0.20 (0.07–0.53)	0.20 (0.07–0.53)	−3.86
Acute respiratory distress syndrome	24	0.47 (0.31–0.70)	0.47 (0.31–0.70)	−1.70	0.44 (0.29–0.67)	0.44 (0.29–0.67)	−1.66	3	0.16 (0.05–0.50)	0.16 (0.05–0.50)	−4.50	0.16 (0.05–0.49)	0.16 (0.05–0.49)	−4.45
Apnoeic attack	6	1.74 (0.78–3.88)	1.74 (0.78–3.88)	−0.66	0.46 (0.20–1.06)	0.46 (0.20–1.06)	−2.30	0	-	-	-	-	-	-
Breath holding	6	1.18 (0.53–2.63)	1.18 (0.53–2.63)	−1.16	1.70 (0.67–4.31)	1.70 (0.67–4.31)	−0.83	1	0.54 (0.08–3.81)	0.54 (0.08–3.81)	−4.45	0.68 (0.09–5.03)	0.68 (0.09–5.03)	−4.17
Infantile apnoea	6	1.15 (0.51–2.56)	1.15 (0.51–2.56)	−1.20	0.46 (0.20–1.06)	0.46 (0.20–1.06)	−2.30	0	-	-	-	-	-	-
Breath sounds abnormal	5	0.28 (0.16–0.67)	0.28 (0.16–0.67)	−3.27	0.29 (0.12–0.71)	0.29 (0.12–0.71)	−3.05	2	0.30 (0.08–1.22)	0.30 (0.08–1.22)	−4.08	0.34 (0.08–1.39)	0.34 (0.08–1.39)	−3.87
Neonatal respiratory depression	4	1.86 (0.70–4.99)	1.86 (0.70–4.99)	−0.98	0.18 (0.07–0.48)	0.18 (0.07–0.48)	−3.87	0	-	-	-	-	-	-
Apparent life-threatening event	2	0.50 (0.13–2.01)	0.50 (0.13–2.01)	−3.43	1.93 (0.37–9.92)	1.93 (0.37–9.92)	−2.04	0	-	-	-	-	-	-
Neonatal respiratory arrest	1	1.77 (0.25–12.7)	1.77 (0.25–12.7)	−3.31	0.53 (0.07–4.22)	0.53 (0.07–4.22)	−4.36	0	-	-	-	-	-	-
Respiratory depth decreased	1	2.37 (0.33–17.1)	2.37 (0.33–17.1)	−3.10	0.80 (0.10–6.66)	0.80 (0.10–6.66)	−3.98	0	-	-	-	-	-	-
Respiratory paralysis	1	1.09 (0.15–7.80)	1.09 (0.15–7.80)	−3.71	0.19 (0.03–1.36)	0.19 (0.03–1.36)	−5.58	0	-	-	-	-	-	-

* Number of AE reports among the total number of tramadol reports. PRR, proportional reporting ratio; CI, confidence interval; ROR, reporting odds ratio; IC, information component; IC_025_, lower limit of the 95% confidence interval of information component. Signals are presented with a shaded background.

**Table 3:** Factors for ACRD of tramadol.

Factors	ACRD Cases (n = 1126)	Non-ACRD Cases (n = 139,595)	*p* Value
Sex (N, %) ^§^			0.0001
Male	438 (42.7%)	49,639 (36.8%)	
Female	589 (57.4%)	85,088 (63.2%)	
Age ^§^			<0.0001
≤17 years	111 (11.7%)	3711 (3.0%)	
18–64 years	624 (65.8%)	83,060 (67.7%)	
≥65 years	213 (22.5%)	35,983 (29.3%)	
Region			<0.0001
Americas	567 (50.4%)	24,494 (17.6%)	
Europe	437 (38.8%)	25,557 (18.3%)	
Asia	86 (7.6%)	86,116 (61.7%)	
Oceania	31 (2.8%)	2332 (1.7%)	
Africa	5 (0.4%)	1096 (0.8%)	
Reporter ^§^			<0.0001
Physician	498 (49.0%)	36,337 (26.0%)	
Pharmacist	160 (15.8%)	14,414 (10.3%)	
Other heath professional	232 (22.8%)	31,545 (22.6%)	
Consumer/Non-healthcare professional	113 (11.1%)	42,305 (30.3%)	
Lawyer	13 (1.3%)	708 (0.5%)	
CYP2D6 inhibitors			<0.0001
Concomitant users	136 (12.1%)	5063 (3.6%)	
Non-concomitant users	990 (87.9%)	134,532 (96.4%)	
Other opioids *			<0.0001
User	350 (31.1%)	9340 (6.7%)	
Non-user	776 (68.9%)	130,255 (93.3%)	
Benzodiazepines			<0.0001
User	223 (19.8%)	3919 (2.8%)	
Non-user	903 (80.2%)	135,676 (97.2%)	<0.0001
Anti-depressant drugs			
User	226 (20.1%)	6403 (4.6%)	
Non-user	900 (79.9%)	133,192 (95.4%)	
Drug abuse			<0.0001
Drug abusers	142 (12.6%)	3248 (2.3%)	
Non-drug abusers	984 (87.4%)	136,347 (97.7%)	
Lethality			<0.0001
Death	233 (20.7%)	3377 (2.4%)	
Survival	893 (79.3%)	136,218 (97.6%)	

The cases included suspect and interacting reports. ^§^ Cases with unknown sex, age, and reporters were excluded (496,717,019 and 14,396 cases, respectively). * The list of other opioids is described in Supplementary Table S3.

**Table 4:** Two-by-two contingency table for disproportionality analysis [49].

Number of Reports	Interest AEs	All Other AEs
Drug of interest	A	B
All other drugs (or opioids)	C	D

The number of reports included in A: both target drugs and specific AEs; B: target drug AEs but with all other AEs; C: specific AEs but with all other drugs; D: all other drugs and all other AEs.

**Table 5:** Formulae and criteria for signal detection [46,47,48].

Indices	Formula	Positive Signal Criteria
PRR	[A/(A + B)]/[C/(C + D)]	PRR ≥ 2
ROR	(A/B)/(C/D)	ROR ≥ 2
IC	IC = log_2_P(AE, Drug)/P(AE)P(Drug)	Lower limit of 95% CI ≥ 0

PRR, proportional reporting ratio; ROR, reporting odds ratio; IC, information component; P, probability; AE, adverse event; CI, confidence interval.

## Data Availability

The datasets analyzed are not publicly available because of the ongoing collection of AE reports. However, they are available from UMC upon reasonable request. Data will be available after approval is obtained from the UMC at https://who-umc.org/ (accessed on 1 March 2023) (request number ER007-2021 and ER-026-2021).

## Supplementary Materials

The following supporting information can be downloaded at https://www.mdpi.com/article/10.3390/ph17020205/s1: Supplementary Table S1: Serious cases associated with acute central respiratory depression; Supplementary Table S2: Detected signal in pediatrics reported acute central respiratory depression; Supplementary Table S3: Frequent concomitant medications in ACRD cases of tramadol and tramadol/paracetamol.
