# Supplementary material for: Risk Factors for Respiratory Depression Associated with Tramadol Based on the Global Pharmacovigilance Database (VigiBase)
Source: Pharmaceuticals (Basel). 2024 Feb 5;17(2):205. doi: 10.3390/ph17020205 (PMC10893455; doi:10.3390/ph17020205)
Supplement: Supplementary file 1 [file pharmaceuticals-17-00205-s001.zip › pharmaceuticals-2822142-supplementary.pdf]

Supplementary Table S1. Serious cases associated with acute central respiratory depression

N (%)	Tramadol (N=938)	Tramadol/paracetamol (N=94)
**Seriousness [a]**		
Caused/Prolonged hospitalization	415 (35.9%)	47 (43.5%)
Life-threatening	232 (20.1%)	28 (25.9%)
Death	382 (33.0%)	26 (24.1%)
Disabling/Incapacitating	14 (1.2%)	0 (0%)
Other	327 (28.3%)	33 (30.6%)
Unknown	224 (19.4%)	16 (14.8%)
**Outcome of serious AEs associated with respiratory depression**		
Died-reaction may be contributory	9 (0.8%)	1 (0.9%)
Died-unrelated to reaction	1 (0.1%)	0 (0%)
Recovered	399 (34.5%)	43 (39.8%)
Recovering	42 (3.6%)	15 (13.9%)
Recovered with sequelae	7 (0.6%)	1 (0.9%)
Not recovered	26 (2.6%)	4 (3.7%)
Fetal	207 (17.9%)	15 (13.9%)
Unknown	229 (19.8%)	29 (26.9%)
**Actions taken to address AEs**		
Drug withdrawn	284 (24.6%)	46 (42.6%)
Dose not changed	26 (2.3%)	2 (1.9%)
Dose reduced	4 (0.4%)	2 (1.9%)
Dose increased	2 (0.2%)	0 (0%)
Not applicable	184 (15.9%)	17 (15.7%)
Unknown	657 (56.8%)	21 (38.0%)
**Outcomes after actions**		
Reaction abated	359 (31.0%)	51 (47.2%)
No effect observed	25 (2.2%)	4 (3.7%)
Effect unknown	166 (14.4%)	11 (10.2%)
Fetal	133 (11.5%)	13 (12.0%)
Not applicable	67 (5.8%)	0 (0%)
Unknown	407 (35.2%)	29 (26.9%)

Serious cases included reports related to respiratory depression regardless of positive signals.

[a] Cases reported with one or more than two kinds of seriousness.

Supplementary Table S2. Detected signal in pediatrics reported acute central respiratory depression

Adverse events	Age group	Tramadol (N=140,721) above full database			
		No. of reports	ROR (CI)	PRR (CI)	IC_{025}
Respiratory arrest	28 days to 23 months	7	14.6 (6.85-31.1)	14.1 (6.79-29.1)	1.65
	2 - 11 years	9	13.6 (7.01-26.3)	13.5 (6.99-25.9)	1.92
	12 - 17 years	8	2.88 (1.43-5.80)	2.88 (1.43-5.78)	0.19
Respiratory depression	28 days to 23 months	11	115.9 (62.4-125)	108.9 (60.8-195)	3.27
	2 - 11 years	7	13.98 (6.61-29.6)	13.88 (6.60-29.2)	1.63
	12 - 17 years	20	13.5 (8.59-21.3)	13.4 (8.56-21.1)	2.60
Bradypnoea	28 days to 23 months	4	148.17 (53.8-408.3)	144.91 (53.7-390.8)	1.35
	2 - 11 years	4	56.93 (20.7-159.9)	56.68 (20.7-155.6)	1.23
	12 - 17 years*	4	15.61 (5.64-43.2)	15.60 (5.64-43.1)	0.80
Hypoventilation	28 days to 23 months	6	48.84 (21.5-110.9)	47.25 (21.4-104.4)	1.99
	2 - 11 years	3	9.29 (2.97-29.03)	9.26 (2.97-28.84)	0.03
	12 - 17 years	4	5.42 (2.01-14.64)	5.41 (2.01-14.60)	0.10

*__Tramadol/paracetamol__: **No. of reports** 3, **PRR** 66.21 (20.6-212.8), **ROR** 65.79 (20.62-209.9), **IC_{025}** 0.62

CI, Confidence Interval

Supplementary Table S3. Frequent concomitant medications in ACRD cases of tramadol and tramadol/paracetamol

Concomitant medications	Tramadol	Tramadol/paracetamol
**Opioids**	**370 (32.0%)**	**16 (14.8%)**
Oxycodone	106 (9.16%)	5 (4.63%)
Morphine	102 (8.82%)	4 (3.70%)
Hydrocodone/paracetamol	79 (6.83%)	1 (0.93%)
Fentanyl	78 (6.74%)	4 (3.70%)
Methadone	44 (3.80%)	1 (0.93%)
Oxycodone/paracetamol	42 (3.63%)	1 (0.93%)
Hydromorphone	41 (3.54%)	0 (0%)
Hydrocodone	25 (2.16%)	0 (0%)
Codeine/paracetamol	22 (1.90%)	3 (2.78%)
**Benzodiazepines**	**257 (22.2%)**	**21 (19.4%)**
Diazepam	85 (7.35%)	6 (5.56%)
Alprazolam	81 (7.00%)	5 (4.63%)
Clonazepam	56 (4.84%)	6 (5.56%)
Lorazepam	45 (3.89%)	1 (0.93%)
Benzodiazepine derivatives	24 (2.07%)	3 (2.78%)
**Pain relievers**	**207 (17.9%)**	**15 (13.9%)**
Paracetamol	121 (10.46%)	7 (6.48%)
Ibuprofen	37 (3.20%)	2 (1.85%)
Diclofenac	30 (2.59%)	2 (1.85%)
Acetylsalicylic acid	29 (2.51%)	6 (5.56%)
Naproxen	28 (2.42%)	1 (0.93%)
**Anesthetics or Hypnotics**	**83 (7.2%)**	**8 (7.4%)**
Zolpidem	54 (4.67%)	8 (7.41%)
Propofol	29 (2.51%)	0 (0%)
**Antidepressants**	**232 (20.1%)**	**19 (17.6%)**
Amitriptyline	53 (4.58%)	3 (2.78%)
Citalopram	48 (4.15%)	0 (0%)
Fluoxetine	33 (2.85%)	1 (0.93%)
Trazodone	33 (2.85%)	1 (0.93%)
Venlafaxine	33 (2.85%)	4 (3.70%)
Sertraline	25 (2.16%)	5 (4.63%)
Duloxetine	24 (2.07%)	5 (4.63%)

Mirtazapine	22 (1.90%)	1 (0.93%)
Bupropion	21 (1.82%)	2 (1.85%)
**Anticonvulsants**	**102 (8.8%)**	**21 (19.4%)**
Gabapentin	67 (5.79%)	3 (2.78%)
Pregabalin	39 (3.37%)	18 (16.67%)
**Muscle relaxants**	**85 (7.3%)**	**8 (7.4%)**
Cyclobenzaprine	41 (3.54%)	5 (4.63%)
Baclofen	23 (1.99%)	3 (2.78%)
Carisoprodol	23 (1.99%)	1 (0.93%)
**Antihistamines**	**51 (4.4%)**	**1 (0.9%)**
Diphenhydramine	32 (2.77%)	0 (0%)
Hydroxyzine	21 (1.82%)	1 (0.93%)
**Others**		
Ethanol	36 (3.11%)	2 (1.85%)
Omeprazole	30 (2.59%)	7 (6.48%)
Quetiapine	26 (2.25%)	3 (2.78%)
Amlodipine	25 (2.16%)	6 (5.56%)
Levothyroxine	24 (2.07%)	1 (0.93%)
Furosemide	22 (1.90%)	6 (5.56%)

Table was presented with concomitant medications for reported more than 20 cases in tramadol and one case reported with none or more than one kind of concomitant medication.

Among 112 ACRD cases ≤17 years, 44.6% were concomitant with other medicines (50 cases), while adults aged over 18 years showed 76.2% of concomitant cases (656 cases) among 861 cases.
